# Vixapatin (VP12), a C-Type Lectin-Protein from *Vipera xantina palestinae* Venom: Characterization as a Novel Anti-angiogenic Compound

**DOI:** 10.3390/toxins4100862

**Published:** 2012-10-18

**Authors:** Tatjana Momic, Gadi Cohen, Reuven Reich, Franziska T. Arlinghaus, Johannes A. Eble, Cezary Marcinkiewicz, Philip Lazarovici

**Affiliations:** 1 School of Pharmacy, Institute for Drug Research, Faculty of Medicine, The Hebrew University of Jerusalem, Jerusalem 91120, Israel; Email: momict@gmail.com (T.M.); gadifco@gmail.com (G.C.); reich@cc.huji.ac.il (R.R.); 2 Center for Molecular Medicine, Department of Vascular Matrix Biology, Excellence Cluster Cardio-Pulmonary System, Frankfurt University Hospital, Frankfurt 60590, Germany; Email: Arlinghaus@med.uni-frankfurt.de (F.T.A.); eble@med.uni-frankfurt.de (J.A.E.); 3 Department of Biology, Temple University College of Science and Technology; Philadelphia, PA 19122, USA; Email: cmarcink@temple.edu

**Keywords:** C-type lectin protein, Vixapatin (VP12), α2β1, integrin, adhesion, migration, tube formation, Matrigel, CAM assay, angiogenesis

## Abstract

A C-type lectin-like protein (CTL), originally identified as VP12 and lately named Vixapatin, was isolated and characterized from Israeli viper *Vipera xantina palestinae* snake venom. This CTL was characterized as a selective α2β1 integrin inhibitor with anti-melanoma metastatic activity. The major aim of the present study was to prove the possibility that this protein is also a potent novel anti-angiogenic compound. Using an adhesion assay, we demonstrated that Vixapatin selectively and potently inhibited the α2 mediated adhesion of K562 over-expressing cells, with IC_50_ of 3 nM. 3 nM Vixapatin blocked proliferation of human dermal microvascular endothelial cells (HDMEC); 25 nM inhibited collagen I induced migration of human fibrosarcoma HT-1080 cells; and 50 nM rat C6 glioma and human breast carcinoma MDA-MB-231 cells. 1 µM Vixapatin reduced HDMEC tube formation by 75% in a Matrigel assay. Furthermore, 1 µM Vixapatin decreased by 70% bFGF-induced physiological angiogenesis, and by 94% C6 glioma-induced pathological angiogenesis, in shell-less embryonic quail chorioallantoic membrane assay. Vixapatin’s ability to inhibit all steps of the angiogenesis process suggest that it is a novel pharmacological tool for studying α2β1 integrin mediated angiogenesis and a lead compound for the development of a novel anti-angiogenic/angiostatic/anti-cancer drug.

## 1. Introduction

Angiogenesis is a complex yet tightly regulated process involving proliferation, migration and capillary sprouting of vascular endothelial cells. Angiogenesis is regulated by a variety of angiogenic growth factors, and ECM proteins and their cognate integrin receptors. The concept that angiogenesis is essential for tumor growth and metastasis has raised interest in the investigation of anti-angiogenic compounds targeting vascular endothelial cells [1]. Therefore, the search for novel angiogenic inhibitors and novel mechanisms of their actions is an important aspect in tumor biology and chemotherapy. Snake venoms are rich biological sources of proteins, which target the cardiovascular system affecting hemostasis [2] and serve as a source for development of novel anti-thrombotic drugs [3]. Furthermore, snake venoms have also been used for purification of different pro-angiogenic [4] and anti-angiogenic [5] factors. These and other factors belong to two protein families—disintegrins [6] and C-type lectin-like proteins (CTLs) [7], which are natural inhibitors of integrin-ligand interactions with relative selectivity towards different integrin α subunit members. C-type lectin-like proteins represent a distinct large family of snake venom proteins. In contrast to C-type lectins, CTLs have lost their carbohydrate-binding function along with their Ca^2+^-binding function, as they have rededicated their carbohydrate recognition loop into a domain swap loop, which typically connects the α and β subunits of CTLs [8,9]. Additionally, these heterodimers are sometimes covalently cross-linked via a disulﬁde bridge. Moreover, CTLs can form oligomeric complexes of heterodimers [10]. In CTLs, the α-subunit (14–15 kDa) and β-subunit (13–14 kDa) are highly homologous. We hypothesize that CTLs are endowed with anti-angiogenic activity; however, this possibility has not been systematically investigated. Interestingly, only members of the CTL family are selective inhibitors against α2β1 integrin, whereas most other integrins are targeted by disintegrins. 

Integrins are a family of receptors on the eukaryotic cell membrane surface, which adhere to multiple ligands and mediate cell-cell and cell-extracellular matrix (ECM) interactions as well as signaling. Integrins and their ligands play key roles in development, proliferation and migration, angiogenesis and tumor progression [11]. The collagen receptor integrin α2β1 is expressed in cells differentiated from all three germ layers: endothelium [12], epithelial cells [13], epidermal keratinocytes cells [14] and fibroblasts [15], platelets [16] as well as ectodermal derived neuronal cells [17]. Moreover, α2β1 integrin is expressed on many tumor cells such as melanoma [18,19], rhabdomyosarcoma [20], ovarian [21] and mammary [22] carcinomas. α2β1 has a key role in angiogenesis [12]. Its activation and differential expression are finely regulated in response to angiogenic factors, such as VEGFs and basic FGF [23]. 

Until now, two other CTLs targeting α2β1 were reported: EMS16 isolated from *Echis multisquamatus *venom [24] and rhodocetin purified from *Calloselasma rhodostoma *venom [25]. More recently a third CTL was isolated from *Vipera xantina palestinae* snake venom and named VP12 [26]. This protein showed potent and selective inhibitory activity against the collagen receptors α2β1. VP12 is a heterodimer protein with an apparent molecular size of 31.7 kDa, composed of two subunits VP12A (15.9 kDa) and VP12 B (15.8 kDa) indicating homology with the C-type lectin-like proteins, EMS16 and rhodocetin. VP12 selectively inhibited melanoma clone adhesion to collagen type I, and reduced melanoma metastasis formation in a mouse model [26,27]. In analogy with the names of other CTLs we named VP12 as Vixapatin. 

In the present study we proved for the first time that Vixapatin is endowed with anti-angiogenic activity, paradigmatically representing an important novel property of this family of CTLs antagonists of α2β1 integrin collagen receptor. We propose Vixapatin as a cellular tool to study angiogenesis and as a lead compound for the development of α2β1 selective drugs with anti-cancer and anti-thrombotic activities. 

## 2. Results and Discussion

### 2.1. Anti-adhesive Properties of Vixapatin

**Figure 1:**
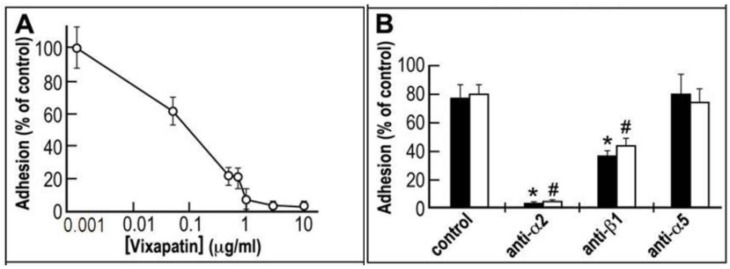
The selective inhibitory effect of Vixapatin on α2-K562 cells adhesion. (**A**) Dose response curve of inhibition of cell adhesion to collagen I; (**B**) Competitive effect of monoclonal antibodies on cell adhesion to immobilized CTLs: EMS16 (black bars) and Vixapatin (white bars), (10 µg/ mL) were immobilized overnight on the plate. The mean number of adherent cells with standard deviation is presented from three independent experiments. *^,#^ *p* < 0.05 compared with the control group.

In an initial functional assay of adhesion, the potency of Vixapatin to inhibit α2β1 integrin was demonstrated using α2K562 transfectants under two different experimental set-ups (Figure 1). In order to verify Vixapatin inhibition of the interaction between collagen I ligand and its receptor α2β1, we first coated the plates with collagen I and measured the effect of different concentrations of Vixapatin on cell adhesion. A typical dose-response adhesion inhibitory curve for Vixapatin was generated and is presented in Figure 1A. With an IC_50_ of 0.1 µg/mL (3.2 nM), Vixapatin effectively inhibited adhesion of α2K562 transfectants, which is similar to EMS16 [24] and rhodocetin [28]. In the second set-up, direct interaction of Vixapatin and α2β1 transfectants was investigated, by immobilizing Vixapatin and as a positive control ESM16 onto plates. Adhesion of α2K562 transfectants to both CTLs was blocked by antibodies directed towards α2 integrin subunit and inhibited to 50% by anti integrin β1 antibodies. An anti-α5 monoclonal antibody failed to block adhesion of Vixapatin, indicating that α5β1 integrin which is constitutively expressed on K562 cells is not involved in the adhesion to Vixapatin (Figure 1B). Control, non-transfected K562 cells did not show any adhesion to CTLs (data not shown). Cumulatively these data indicate that Vixapatin recognized the integrin α2 subunit, in line with additional data showing that the recombinant collagen-binding A-domain of α2 integrin binds to Vixapatin [29] similar to EMS16 [24] and rhodocetin [28].

### 2.2. Effect of Vixapatin on Proliferation of HDMEC

**Figure 2:**
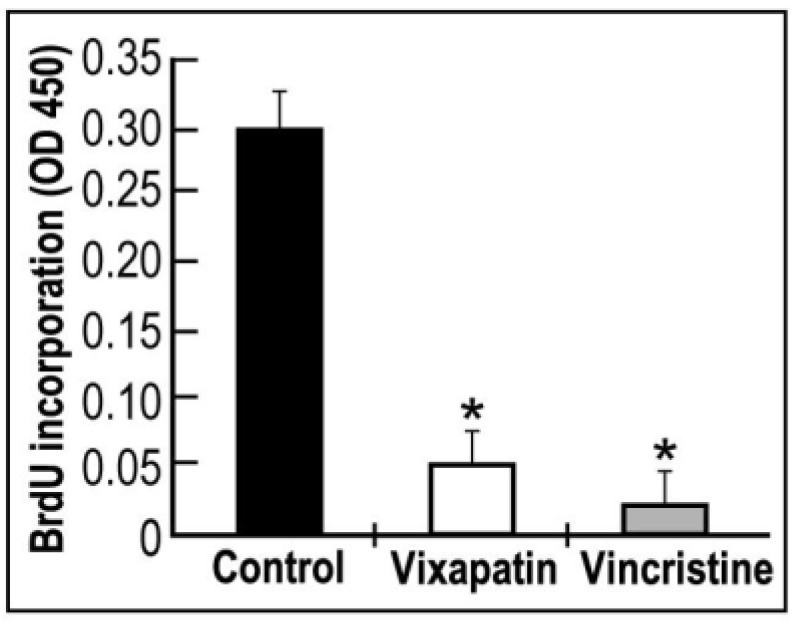
Effect of Vixapatin on proliferation of HDMEC. 1 µM Vixapatin and 3.6 µM vincristine (positive control) were used, and proliferation was measured by BrdU proliferation assay. Values are mean ± SD (*n* = 3).* *p * < 0.05 compared with the control group.

The proliferation of endothelial cells from a pre-existing capillary is an important step in the angiogenic effect [30]. Therefore, an angiostatic compound should inhibit this process. To verify this possibility we investigated the effect of Vixapatin on human dermal microcapillary endothelial cells (HDMEC) proliferation using BrdU assay. A significant 84% decrease in cell proliferation was observed similar to the effect of the anti-mitotic drug, vincristine (Figure 2). 

### 2.3. Effect of Vixapatin on Cell Migration

**Figure 3:**
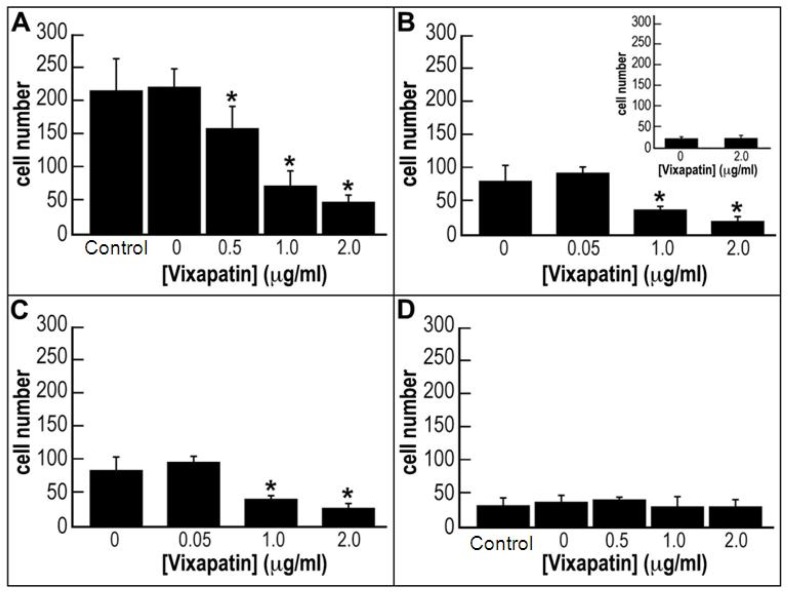
Vixapatin inhibition of HT1080 cells and MDA-MB-231 cell migration. Migration of: (**A**) HT1080 cells across the filters coated with collagen I, (**B**) HT1080 cells across the filters coated with collagen IV; (**C**) MDA-MB-231 cells across the filters coated with collagen I; (**D**) MDA-MB-231 cells across the filters coated with collagen IV, in the presence of different concentrations of Vixapatin. Vixapatin was used as chemoattractant and was added to the lower chamber, while the cells were in the upper chamber and the filter coated with respective collagens. Inset: lack of cell migration when Vixapatin and HT1080 cells were applied in the upper chamber. The data are presented as the mean number of migrating cells with SD from three independent experiments. * *p *< 0.05 compared with the control group.

Since α2β1 integrin is expressed in different tumors and enhances their angiogenesis [31,32] we investigated the effect of Vixapatin on several tumor cell lines. We investigated Vixapatin chemoattractant activity onto human fibrosarcoma HT1080 cells and human breast cancer MDA-MB-231 cells using a Boyden chemotaxis chamber. We used type I and IV collagen as immobilized substrata and observed that cell migration on collagen I was three times higher than on collagen IV, in line with higher affinity of α2β1 integrin for collagen I [33]. To test the effect of Vixapatin on cell migration, two set-ups of experiments were done. First, Vixapatin was added to the lower chamber in DMEM containing 0.1% BSA. Acute stimulation of HT1080 cells with different concentrations of Vixapatin resulted in similar (*p* < 0.05 compared with the control group = absence of Vixapatin) dose-dependent decrease of cells, which migrated across filters coated with either collagen I or collagen IV (Figure 3A,B). An apparent IC_50_ of 25 nM was determined. In the second experimental approach (Figure 3B, Inset) the HT1080 cells and Vixapatin were added together in the upper chamber on the top of filters coated with collagen IV. The experiment clearly indicated the lack of cell migration to the lower chamber indicative of specificity of cell migration. The first experimental set-up was also done with MDA-MB-231 cells. The dose-dependent inhibition of the number of migrated cells was obtained only with collagen I coated filters (Figure 3C). In case of collagen IV, no effect of Vixapatin on cell migration was noticed (Figure 3D). Although it is well known that HT1080 [34] and MDA-MB-231 [35] express α2β1 integrin, the relative quantitative levels of α1β1 and α2β1 in these cells are unknown, therefore we can not exclude the possibility that decreased migration of MDA-MB-231 cells is also a reflection of the reduced number of integrin receptors. In conclusion, it is reasonable to claim that Vixapatin slightly inhibited the α2β1-mediated migration of MDA-MB-231 cells on collagen IV, an effect which was not observed on collagen I. The Vixapatin-induced inhibition of migration was also found with fibrosarcoma HT1080 cells by monitoring the increase of impedance values over time as HT1080 cells migrated along a haptotactic gradient of collagen covering the electrode [29]. The ability of Vixapatin to inhibit cell migration further characterizes Vixapatin as an angiostatic compound. 

### 2.4. Effect of Vixapatin on Tube Formation in Matrigel Assay

**Figure 4:**
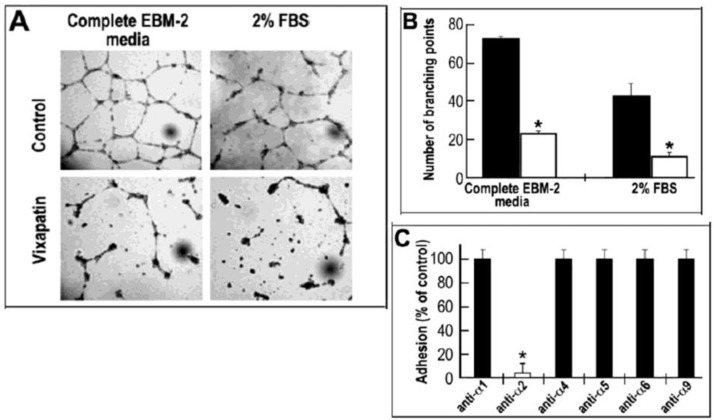
The inhibitory effect of Vixapatin on HAEC tube formation in Matrigel assay. (**A**) The tube formation in Matrigel was induced by complete EBM-2 medium (left side) or 2% FBS (right side) in the absence (first row) or in the presence (second row) of 1 μM Vixapatin; (**B**) Graphic values of number of branching points, counted per observation field in the absence (control, black bars) and in the presence (white bars) of 1 μM Vixapatin; (**C**) Competitive effect of Vixapatin on cell adhesion to immobilized monoclonal antibodies directed towards different integrin α subunits. The mean numbers of adherent cells with SD are shown from three independent experiments. * *p* < 0.05 compared with the control group.

Processes necessary for new vessel formation include the migration of endothelial cells from the existing capillaries and their morphogenic rearrangement into a tube like structure [36]. In order to investigate Vixapatin’s ability to inhibit tube formation, its effect on microvascular human aortic endothelial cells (HAEC) was investigated in a Matrigel angiogenesis assay. A significantly decreased number of newly formed capillaries were observed in the presence of 1 µM Vixapatin. This inhibitory effect was evident both in the presence of conditioned endothelial cell medium (EBM-2) as well as 2% FBS (Figure 4A). The number of branching points during tube formation in the presence and absence of Vixapatin are presented in Figure 4B. Vixapatin significantly reduced the number of branching points by 75% in both conditions. Using monoclonal antibodies directed towards different α integrin subunits in adhesion assays, we confirmed that Vixapatin selectively inhibited the adhesion of the cells to the monoclonal α2 integrin subunit expressed on HAEC (Figure 4C). These data indicated that collagen I-α2 integrin interaction of the cells is involved in angiogenesis, as previously described with lung carcinoma cells [37]. These findings proposed Vixapatin as an anti-angiogenic compound. 

### 2.5. Effect of Vixapatin on bFGF Induced Physiological Angiogenesis in the CAM Quail Embryonic Model

**Figure 5:**
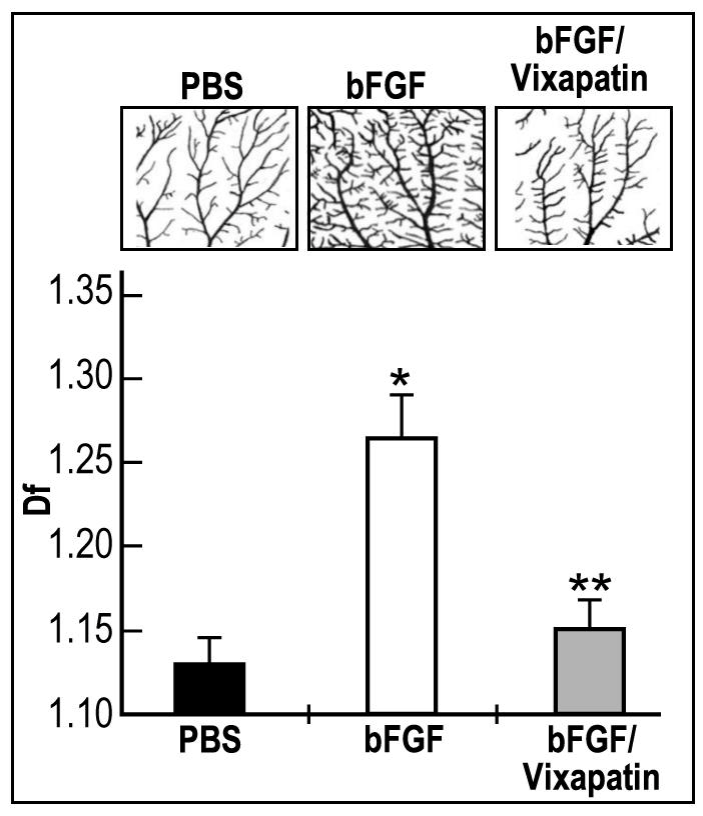
The inhibitory effect of Vixapatin on bFGF induced angiogenesis in the Japanese quail CAM assay. Representative binary images of mid-arterial end points fragments of CAMs dissected from embryos are presented on the upper panels: basal level of angiogenesis (PBS); angiogenesis induced by 50 ng/mL bFGF (bFGF); the inhibitory effect of 1 µM Vixapatin on angiogenesis induced by bFGF (bFGF/Vixapatin). Fractal dimension (*D*_f_) representing the angiogenic effect is presented in the lower panel. The fractal dimension values ± SD are shown from three independent experiments. * *p* < 0.05 compared with PBS. ** *p *< 0.001 compared with bFGF.

The quail chorioallantoic membrane (CAM) assay is a good model to measure and quantify bFGF angiogenic effects [38]. Therefore, we considered this assay to evaluate the potential inhibitory effect of Vixapatin on bFGF-induced angiogenesis. The binary images of the mid-arterial endpoint fragments of CAMs showed a significant overgrowth of small capillaries after 24 h of treatment with bFGF in comparison with control vehicle (PBS) treated embryos (Figure 5, upper panel-bFGF). 1 µM Vixapatin potently inhibited bFGF-induced capillary formation by 70%, thereby approaching the levels of the bFGF-free control (Figure 5). This finding supports the proposal that Vixapatin is an anti-angiogenic factor.

### 2.6. Effect of Vixapatin on C6-Induced Pathological Angiogenesis in the CAM Quail Embryonic Model

**Figure 6:**
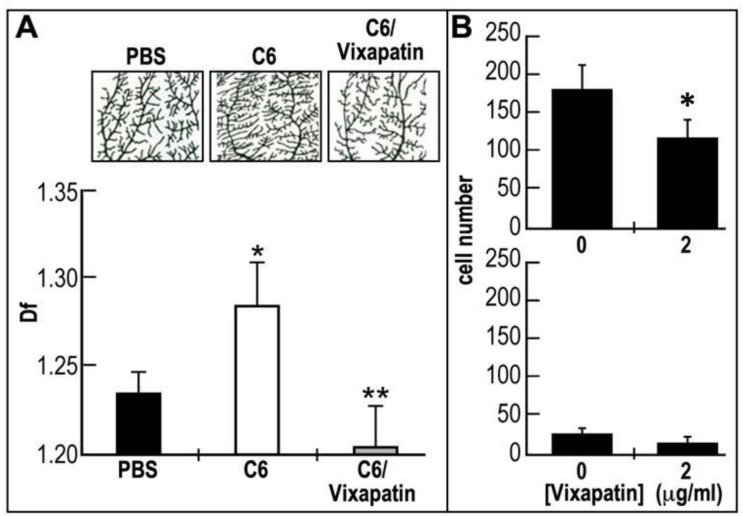
The inhibitory effect of Vixapatin on pathological angiogenesis induced by C6 cell line in shell-less embryonic quail CAM system. (**A**) Representative binary images of mid-arterial end points fragments of CAMs dissected from embryos are presented on the upper panels: basal angiogenesis (PBS), pathological angiogenesis induced by C6 rat glioma cell line (C6), C6 induced angiogenesis in the presence of 1 μM Vixapatin (C6/Vixapatin). Lower panel: graphic values of angiogenesis expressed by fractal dimension (*D*_f_). *p* < 0.05 compared with PBS. ** *p* < 0.001 compared with C6; (**B**) The effect of Vixapatin on migration of C6 cells in Boyden chamber. Upper panel: Migration of the cells across the filters coated with collagen I. Vixapatin was used as chemoattractant and was added to the lower chamber, while the cells were in the upper chamber. Lower panel: lack of inhibition of Vixapatin of cell migration toward filters coated with collagen IV, when applied in the upper chamber. *p* < 0.05 compared with the control (in the absence of Vixapatin).

C6 glioma tumor cells injected under the CAM is an useful assay to measure pathological angiogenesis [39]. Therefore, we sought to investigate the potential inhibitory effect of Vixapatin on this model. To achieve this aim we injected C6 glioma cells in shell-less embryonic CAM system and measured the effect of the tumor cells on mid-arterial capillary sprouting (Figure 6A). In this system, C6 cells induced a strong angiogenic effect most probably due to release of VEGF [40]. This is represented by a significant increase in angiogenesis expressed by increased fractal dimension (*D*_f_) values from 1.12 to 1.26. The upper panel in Figure 6A clearly indicated the sprouting of small branching capillaries from the arterial tree. Treatment with 1 μM Vixapatin, a concentration found not cytotoxic to the cells, reduced the C6 induced angiogenic effect by 95%. This finding suggests that α2β1 integrin is involved in C6 tumor-induced angiogenesis and that Vixapatin has a potent anti-angiogenic effect. In addition, we investigated the effect of Vixapatin on migration of C6 cells in Boyden chamber assay *in vitro*, using the same set-ups described in Figure 3. At a concentration of 65 nM, Vixapatin inhibited the migration of cells towards collagen I by 35%, while the migration of the cells towards collagen IV was poor and not significantly affected (Figure 6B). These findings further support the anti-angiogenic effects of Vixapatin measured in the CAM assay. 

### 2.7. Discussion

In this study we characterized for the first time the anti-angiogenic activities of Vixapatin (VP12), a novel C-type lectin-like protein with selective α2β1 integrin inhibitory activity [26]. Vixapatin showed potent anti-angiogenic/angiostatic activity *in vitro*, observed by inhibition of α2K562 cells adhesion, endothelial cell proliferation, tube formation in Matrigel assay and cell migration, as well as *in vivo* towards basic FGF and glioma-induced angiogenesis in the chorioallantoic membrane assay. The inhibitory effect of Vixapatin on these major steps in the angiogenic process further confirmed the contribution of α2β1 integrin in angiogenesis [37] as well as confirming our hypothesis that Vixapatin may serve as a pharmacological tool for studying α2β1 integrin mediated angiogenesis. VP12 by virtue of its antagonistic effect on α2β1 integrin was also found as an anti-thrombotic agent inhibiting collagen I induced platelet aggregation [29]. Platelet activation is accompanied by the release of microparticles which induce angiogenesis both *in vitro* and *in vivo* [41]. Therefore by inhibiting platelet activation, VP12 is supposed to generate in whole animals a stronger anti angiogenic effect mediated by both its direct α2β1 integrin antagonism and indirectly by inhibiting platelets-induced angiogenesis.

Many studies, using both direct and indirect approaches proposed the concept of α2β1 integrin involvement in angiogenesis. Niewirowska *et al.*, 2011 [42] reported that lumican, a small leucine-rich proteoglycan that binds to the α2 integrin I domain, is an efficient inhibitor of cell adhesion and migration and affects angiogenesis both by interfering with α2β1 receptor activity and down regulating proteolytic activity associated with surface membranes of endothelial cells. In other studies endorepellin, the COOH-terminal domain of the heparin sulfate proteoglycan perlecan, was found to inhibit several aspects of angiogenesis [43] being characterized as an angiostatic compound [44]. Aggretin, a collagen-like α2β1 agonist purified from *Calloselasma rhodostoma *snake venom, was shown to increase human umbilical vein endothelial cell proliferation and migration therefore inducing angiogenesis [45]. VEGF, the most potent angiogenic factor, regulates the induction of α1β1 and α2β1 expression by endothelial cells, an effect considered an important angiogenic mechanism [12]. Angiocidin, a protein isolated from lung carcinoma exerts its anti-tumor and anti-angiogenic activities by antagonizing α2β1/collagen adhesive interactions [45]. The recombinant two kringle domain of human tissue-type plasminogen activator (TK1-2) has been shown to inhibit endothelial cell proliferation, angiogenesis, and tumor cell growth by interfering with integrin α2β1 [47]. These studies emphasize that agonists of α2β1 integrin promote angiogenesis while antagonist of α2β1 are anti-angiogenic/angiostatic compounds. 

Snake venoms are considered a rich source of naturally occurring inhibitors of α2β1 integrin, which belong to the family of C-type lectin-like proteins. So far, three of them have been identified: rhodocetin from *Calloselasma rhodostoma* [25], EMS16 from *Echis multisquamatus* [24] and VP12 from *Vipera xantina palestinae* [26]. The present research characterizing Vixapatin (VP12) as an anti-angiogenic compoud is the first anti-angiogenic study with a member of CTL snake venom family. It is proposed that other members are endowed with anti-angiogenic/angiostatic activity. In the light of recent studies, which are emphasizing the crucial role of α2β1 integrin in angiogenesis [23,48], we propose Vixapatin as a novel cellular tool for studying α2 integrin mediated angiogenesis. The anti-angiogenic activity of Vixapatin suggests that further investigation of the same effect of other members of CTLs which specifically interact with α2β1 integrin, may enlarge the arsenal of anti-angiogenic compounds based on snake venom CTLs. 

## 3. Experimental Section

### 3.1. Materials

Collagen IV (from bovine placenta villi) was purchased from Chemicon (Temecula, CA, USA), and collagen I (from rat tail) and Matrigel from BD Biosciences (Bedford, MA, USA). 96-well polystyrene EIA/RIA plates were obtained from Nunc (Roskilde, Denmark). The integrin antibodies anti-α1, anti-α2, anti-α4, anti-α5, anti-α6, anti-α9 were purchased from BD Bioscience (San Jose, CA, USA). Anti-β1 monoclonal integrin antibody was obtained from Chemicon (Temecula, CA, USA). Bovine serum albumin (BSA), Hank’s Balanced Salt Solution (HBSS) and Vincristine sulfate were purchased from Sigma-Aldrich (St. Louis, MO, USA). CellTracker™ Green 5-Chloromethylfluorescein Diacetate (CMFDA), was purchased from Invitrogen-Molecular Probes (Eugene, OR, USA). 5-bromo-2'-deoxyuridine (BrdUrd) kit was obtained from Roche (Mannheim, Germany). 13-mm polycarbonate filters (8 μm pore-size, polyvinyl pyrrolidone-free) were purchased from Nucleopore, Whatman (Maidstone, United Kingdom). Diff Quik kit was purchased from Dade Diagnostics (Aguada, Puerto Rico). Basic fibroblast growth factor (bFGF) was purchased from CytoLab (Rehovot, Israel). Fertilized Japanese quail (*Coturnix coturnix japonica*) eggs were purchased from Boyd’s Bird Co (Pullman, WA, USA).

### 3.2. C-Type Lectin-Like Proteins

Snake venom C-type lectin-like (CTL) protein Vixapatin was purified from *Vipera xantina palestinae* snake venom as previously described [26]. Snake venom C-type lectin-like proteins EMS16 was isolated from *Echis multisquamatus* venom as previously described [24].

### 3.3. Cell Lines

Primary human dermal microvascular endothelial cells (HDMEC) cultured in complete endothelial cell basal media-2 (EBM-2) were purchased from Cambrex (Walkersville, MD, USA). Primary endothelial cells were used in experiments between 5 and 8 passages. Human aortic endothelial cells (HAEC) were kindly provided by Prof. Peter I. Lelkes, Drexel University and cultured as previously described [49]. Rat glioma C6 cell line (ATCC, Biological Industries, Israel), was cultured in F-12K medium supplemented with 2.5% FBS and 15% horse serum. Human fibrosarcoma HT-1080 cells were cultured in Eagle’s Minimum Essential Medium supplemented with 10% FBS, and human breast carcinoma MDA-MB-231 cells in Leibovitz’s L-15 Medium with 10% FBS (ATCC, Biological Industries, Israel). K562 cells transfected with α1 and α2 integrin subunits [26] and cultured in RPMI 1640 supplemented with 10% FBS and 0.5 mg/mL of G418. 

### 3.4. Cell Adhesion Studies

Adhesion assays were carried out essentially as described previously with minor changes [50]. Before the day of the experiment, each well of a 96-well plate was coated with 10 µg/mL CTLs or monoclonal integrin antibodies in buffered saline (PBS) and incubated overnight at 4 °C. When plates were coated with 10 µg/mL collagen I (α2β1 ligand) or 1 µg/mL collagen IV (α1β1 ligand), 0.02 M acetic acid was used instead of PBS. Thereafter non-specific binding was blocked by incubating the wells with 1% (*w*/*v*) bovine serum albumin (BSA) in Hank's Balanced Salt Solution (HBSS) containing 5 mM MgCl_2_, at room temperature for 1 h prior to use. Cells were labeled by incubation with 12.5 µM CMFDA in HBSS without 1% BSA at 37 °C for 30 min. The labeled cells were then centrifuged at 1000 rpm and washed twice with HBSS containing 1% BSA to remove excess CMFDA. Labeled cells (1 × 10^5^ cells/well) were added to each well in the absence or presence of inhibitor and incubated at 37 °C for 60 min. After prior incubation with inhibitor for 30 min at 37 °C, cells were added to the wells. Unbound cells were removed by washing the wells three times with 1% (*w*/*v*) BSA in HBSS, and bound cells were lysed by the addition of 0.5% Triton X-100 (diluted in DDW). The amount of fluorescence in each well was quantified with a SPECTRAFluor Plus plate reader (Tecan), at *λ*_ex_ = 485 nm and *λ*_em_ = 530 nm. To determine the number of adhered cells from the fluorescence values, a standard curve was generated by serial dilutions of known numbers of CMFDA-labeled cells. 

### 3.5. Cell Proliferation Assay

The HDMEC proliferation assay was performed using (BrdUrd) kit according to the manufacturer’s instructions [51]. 

### 3.6. Cell Migration Assay

Cell migration was determined in a modified Boyden chamber as described previously [52]. 13-mm polycarbonate filters were coated with 50 µg/filter either collagen IV or collagen I, dissolved in 0.5 N acetic acid. The proteins were allowed to dry for 1h, at 37 °C on the filters. Two experimental set-ups were done. In the first experimental set-up Vixapatin was used as chemoattractant and was added to the lower chamber in DMEM containing 0.1% BSA. The cells were harvested by brief exposure to 1 mM ethylenediaminetetraacetic acid (EDTA), washed with DMEM containing 0.1% BSA, and 2 × 10^5 ^cells in 0.2 mL were added to the upper chamber. The cells were incubated for 6 h at 37 °C in humidified atmosphere of 95% air and 5% CO_2._ In the second set-up of experiments Vixapatin was added together with the cells in the upper chamber and cell migrated in response to the conditioned medium as nonspecific chemoattractant. The cells that migrated across the filter and attached to its lower surface were stained with Diff Quik kit, photographed and counted in five randomized fields. 

### 3.7. Human Aortic Endothelial Cells Tube Formation in Matrigel Assay

The assay was performed using a 96-well plate coated with growth factor reduced Matrigel. Briefly, 1 × 10^4^ human aortic endothelial cells (HAEC) per well (in complete EMB-2 media or 2% FBS) were added in the presence or absence of Vixapatin, and the plate was incubated overnight at 37 °C in 5% CO_2_. Images were captured under an inverted microscope (Olympus IX81) with 35× magnification. Images were transferred to ImageJ software and number of branching points was counted per observation field. 

### 3.8. Angiogenesis in Chorioallantoic Membrane (CAM) Quail Embryonic Model

The assay of bFGF induced and tumor induced angiogenesis in the quail embryonic CAM system was performed as described previously [38,53,54]. Fertilized Japanese quail eggs were cleaned with ethanol, and maintained at 37 °C until embryonic day three in incubator without CO_2_. The shells were then opened with a razor blade and sterile scissors the contents transferred into 6-well tissue culture plates and returned to the 37 °C incubator. At embryonic day seven, the compounds in sterile conditions were topically applied on the surface of the CAM and after 24 h the effect on the aortic tree was evaluated. Embryos were divided into experimental groups, each containing at least 10 embryos. The control group received a vehicle (PBS) treatment. In the experiments of C6 induced tumors under the CAM, C6 glioma cells (1 × 10^7^/50 μL) were injected at day seven under the CAM and tumor induced angiogenesis was measured at day 12. At the end of the experiment the embryos were fixed with 5 mL of pre-warmed 2% gluteraldehyde, 4% paraformaldehyde in PBS for 48 h at room temperature. The membranes with and without tumor were dissected and transferred onto the glass slide. The membranes were mounted onto glass slides for evaluation of fractal dimension (*D*_f_) as described earlier [38]. The area of CAM selected as a square for analysis of vascularization ratio, was localized as previously described [38] or in the case of the tumor induced angiogenesis, in the opposite site to the tumor on the membrane. For example, if tumor was developed in the right corner of the CAM, the vascularization tree for analysis was framed in the left corner of membrane [53]. 

### 3.9. Statistics

Where appropriate, all data are expressed as mean ± SD (*n *= 6–16) from three to six independent experiments. Comparisons between groups were analyzed by Student’s t-test or one-way analysis of variance (Microsoft Office Excel, 2007) to determine significant differences. *p* values less than 0.05 were considered statistically significant.

## 4. Conclusions

Vixapatin (VP12) is known as a C-type lectin-like protein isolated from *Vipera xantina palestinae* venom. The present study attributes a novel anti-angiogenic/angiostatic property to Vixapatin. It is proposed that this property is the result of the inhibitory effect of Vixapatin on α2β1 integrin mediated angiogenic effects in endothelial cells. This novel activity of Vixapatin, in conjunction with its anti-melanoma property, supports the proposal that this snake venom derived CTL may be used as a pharmacological tool for investigations in cancer research. 
